# The Employment of Midwives by Medical Men

**Published:** 1898-11-12

**Authors:** 


### The Employment of Midwives by Medical Men.

The difficulty which general practitioners have lately experienced in obtaining the services of good and capable qualified assistants to take the places of the unqualified men who had been employed until the celebrated "notice," issued by the General Medical Council last November, rendered their further employment impossible, brings into renewed prominence the question of the relation which ought to exist between medical practitioners and midwives. Anyone who knows anything about general practice knows how it gathers around the midwifery, and how important this branch of work is to all who wish to do family practice. But everyone also knows how trying the work is, how urgent and irregular are its calls, and how difficult it is to meet them when working single-handed. It is this, indeed, which has been at the root of the trouble. Men who did not want, and could not afford to pay for, qualified assistance, wanted, and still want, someone to be at hand to attend urgent midwifery calls, and there can be no doubt that if the bite of the General Medical Council should prove as bad as its bark, and if the "notice" should be put in force in accordance with its literal terms, a very large amount of midwifery must be given up by medical practitioners and must fall into the hands of midwives.

This is a very serious matter for the rank and file of the medical profession, and we cannot but think that they have been dealt with somewhat unfairly by the Medical Council, not in regard to the abolition of unqualified practice, but in regard to the vagueness and uncertainty which is still left hanging over the question, how far a practitioner may legitimately go in the employment of unqualified assistance. In the "notice" it is distinctly stated that any practitioner who is proved to have employed an unqualified assistant "to attend or treat patients in respect of matters requiring professional discretion or skill" is liable to have his name erased from the Register. This would seem clear; but in the same notice it is added that "the foregoing notices do not apply so as to restrict . . . the legitimate employment of . . . dressers, midwives, dispensers, and surgery attendants, under the immediate personal supervision of Registered Medical Practitioners." What, then, is the "legitimate" employment of a midwife? Now, to the ordinary common-sense man, it would seem pretty clear that the employment of midwives "under the immediate *personal* supervision of Registered Medical Practitioners" would hardly be likely to help doctors much in their daily work, and we are bound to believe that, however strictly the Council intends to interpret its "notice" in regard to assistants, it must have different views in regard to midwives, and, indeed, must be prepared to allow considerable latitude in the interpretation of the words, "under the immediate supervision," &c., so far as they apply to midwives. The pity is that the Council does not definitely say so, for at the present time many practitioners feel themselves much aggrieved by the existing uncertainty on this question. They have loyally given up their unqualified assistants. May they, then, employ midwives? In other words: Is it a "legitimate employment" of midwives for a medical practitioner to engage one or several of them to attend his ordinary cases just in the same way as certain maternity charities do? The fact of these great maternities being charities does not touch the question. They are, in fact (putting on one side the question of payment), great midwifery practices, in which the difficult cases are attended to by qualified medical men—men of the highest professional rank—while the ordinary cases are attended by their unqualified female assistants. Now these unqualified assistants, or midwives, undoubtedly attend the cases without any personal supervision whatever from qualified medical officers, and the general practitioners may well ask whether they may not do in their practices what is done every day in the practices of the great maternity charities? If they can, not only is all real hardship in the "notice" about unqualified assistants done away with, for it is only in regard to midwifery that the difficulty arises, but a way would seem to be opened by which to solve the great and difficult question of the midwives. To this, however, we must refer in a future article.

